# Using magnetic resonance imaging to map the hidden burden of muscle involvement in systemic sclerosis

**DOI:** 10.1186/s13075-022-02768-z

**Published:** 2022-04-11

**Authors:** Laura Ross, Anniina Lindqvist, Benedict Costello, Dylan Hansen, Zoe Brown, Jessica A. Day, Wendy Stevens, Andrew Burns, Warren Perera, Marcus Pianta, André La Gerche, Mandana Nikpour

**Affiliations:** 1 Department of Medicine, The University of Melbourne at St Vincent’s Hospital Melbourne, 41 Victoria Pde, Fitzroy, VIC 3065 Australia; 2 Department of Rheumatology, St Vincent’s Hospital, Fitzroy, VIC Australia; 3 Sports Cardiology Laboratory, Baker Heart and Diabetes Institute, Melbourne, VIC Australia; 4 Department of Cardiology, St Vincent’s Hospital, Fitzroy, VIC Australia; 5 Inflammation Division, Walter and Eliza Hall Institute, Parkville, VIC Australia; 6 Department of Radiology, St Vincent’s Hospital, Fitzroy, VIC Australia

**Keywords:** Systemic sclerosis, Myopathy, Myositis, Magnetic resonance imaging

## Abstract

**Background:**

Skeletal muscle can be directly affected by systemic sclerosis (SSc); however, a significant burden of SSc-associated myopathy is undetected because clinical parameters such as weakness and creatine kinase (CK) are unreliable biomarkers of muscle involvement. This study presents qualitative and quantitative magnetic resonance imaging (MRI) findings that quantify the prevalence of myopathy and evaluate any association between skeletal and cardiac muscle involvement in SSc.

**Methods:**

Thirty-two patients with SSc who fulfilled the 2013 American College of Rheumatology/European League Against Rheumatism classification criteria underwent skeletal muscle MRI in addition to cardiac MRI. Skeletal muscles were independently assessed by two musculoskeletal radiologists for evidence of oedema, fatty infiltration and atrophy. Skeletal muscle T2 mapping times and percentage fat fraction were calculated. Linear regression analysis was used to evaluate the clinical and myocardial associations with skeletal muscle oedema and fatty infiltration. Cardiac MRI was performed using post gadolinium contrast imaging and parametric mapping techniques to assess focal and diffuse myocardial fibrosis.

**Results:**

Thirteen participants (40.6%) had MRI evidence of skeletal muscle oedema. Five (15.6%) participants had fatty infiltration. There was no association between skeletal muscle oedema and muscle strength, creatine kinase, inflammatory markers or fibroinflammatory myocardial disease. Patients with skeletal muscle oedema had higher T2-mapping times; there was a significant association between subjective assessments of muscle oedema and T2-mapping time (coef 2.46, *p* = 0.02) and percentage fat fraction (coef 3.41, *p* = 0.02). Diffuse myocardial fibrosis was a near-universal finding, and one third of patients had focal myocardial fibrosis. There was no association between skeletal myopathy detected by MRI and burden of myocardial disease.

**Conclusions:**

MRI is a sensitive measure of muscle oedema and systematic assessment of SSc patients using MRI shows that myopathy is highly prevalent, even in patients without symptoms or other signs of muscle involvement. Similarly, cardiac fibrosis is highly prevalent but occurs independently of skeletal muscle changes. These results indicate that novel quantitative MRI techniques may be useful for assessing sub-clinical skeletal muscle disease in SSc.

## Introduction

Systemic sclerosis (SSc) is a multi-system disease characterised by widespread fibrotic, vascular and inflammatory phenomena [1]. SSc-associated myopathy (SM) has long been appreciated but remains poorly understood and ill-defined. The prevalence of SM varies from 5 to 96% [2, 3], and inflammatory, fibrotic, necrotic, vasculopathic and metabolic muscle changes have been reported without any clearly definable histopathological signature [4]. Clinical definitions of SM include various combinations of proximal weakness, myalgia, elevated muscle enzymes, electromyography and histopathological changes [5–11]. Myopathy, irrespective of how it is defined, is associated with increased disability and mortality in SSc [6, 8, 12]. An important clinical association between SM and SSc-associated heart involvement (SHI) has been observed [13].

Manual muscle testing (MMT) and creatine kinase (CK), along with patient reported outcomes have been recommended as outcome measures in clinical trials of SM [3]. However, CK unreliably detects SM, with only 67% of patients referred for muscle biopsy recording an elevated CK [4, 14]. Clinical evaluation of muscle involvement using MMT is fraught given the unquantified effects of concurrent skin thickening, joint contracture and treatment-induced myopathy. Additionally, MMT cannot distinguish muscle inflammation from residual weakness due to long standing muscle disease and damage as well as the potential effects of disuse atrophy [15]. Consideration needs to be given to more reliable methods of distinguishing active SM, that is potentially responsive to treatment, from muscle damage, which by definition is irreversible [3].

Skeletal muscle magnetic resonance imaging (sMRI) is an appealing diagnostic modality as it is non-invasive and can identify the presence of myositis, delineate its extent and assess treatment response [15–17]. sMRI can visualise diverse muscle pathologies including oedema, suggesting active inflammation, as well as fatty replacement and muscle atrophy indicating disease chronicity and damage [18]. There is increasing interest in patterns of muscle involvement to narrow differential diagnoses and identify subtypes of idiopathic inflammatory myopathy (IIM). Distinct patterns have been described for inclusion body myositis, anti-signal recognition particle (SRP) positive necrotising myositis and particular muscular dystrophies, and preliminary evidence suggests that the radiological pattern of juvenile dermatomyositis is of prognostic importance [15, 16, 18–20].

One limitation of sMRI in the assessment of myopathy is the lack of standardised scoring systems. Visual assessment scales that semi-quantitatively evaluate the burden of muscle atrophy and fatty infiltration on T1-weighted images and muscle oedema on either short tau inversion recovery (STIR) or T2-weighted sequences have been developed and validated for hereditary myopathies [21–24]. However, there is only fair-to-moderate correlation between scorers when such scales are applied to IIM [19, 21]. Quantitative measures of skeletal myopathy are emerging, namely T2-mapping, as a measure of oedema, and fat fraction (FF) to quantify fatty muscle infiltration and measure damage [25]. Quantitation of T2-mapping times, muscle volume and FF have recently been shown to correlate with radiologist assessment scores, muscle strength and distinguish IIM patients from healthy controls and patients with muscular dystrophy [26, 27]. The role of T2-mapping and FF calculations in SSc has not been evaluated.

The aim of this study was to quantify the radiological burden of SM, using the current reference standard of expert visual assessment as well as novel quantitative sMRI techniques. Additionally, we evaluated the clinical associations of radiological muscle oedema and damage, including with changes of cardiac structure and function.

## Methods

### Study population

Consecutive adult patients (age > 18 years) who fulfilled the 2013 American College of Rheumatology/European League Against Rheumatism classification criteria for SSc [28] with a disease duration of < 4 years or > 10 years were invited to participate, to ensure recruitment across the spectrum of SSc disease duration. Disease onset was defined by the presence of the first non-Raynaud’s symptom. Patients were ineligible if they had a history of cardiac disease, including myocarditis, ischaemic heart disease or pulmonary arterial hypertension, renal impairment (estimated glomerular filtration rate < 40 mL/min/1.73 m^2^) or contraindication to MRI. Patients were recruited irrespective of their history of musculoskeletal manifestations of SSc. All patients were classified into limited or diffuse (dcSSc) subsets according to LeRoy criteria [29]. SSc disease manifestations and autoantibodies were defined as present if ever recorded during follow-up. All patients gave written informed consent to participate. Ethics approval was granted by St Vincent’s Hospital, Melbourne Human Research Ethics Committee (HREC 181/18), and the study was performed in accordance with the Declaration of Helsinki.

### Data collection

Demographic and clinical data were recorded. Clinical examination included measurement of the modified Rodnan Skin Score (mRSS) and proximal muscle strength by MMT. Muscle weakness was considered present if muscle strength was ≤ 4/5, where 5 denotes normal muscle strength. Blood testing was performed, including measurement of CK, erythrocyte sedimentation rate (ESR) and C-reactive protein (CRP). SSc-specific, myositis-specific (MSA) and myositis-associated antibody (MAA) testing was performed. SSc-specific autoantibody testing included measurement of anti-centromere, anti-topoisomerase I, RNA polymerase III, anti-Ku, PMScl100, PMScl70, anti-fibrillarin, anti-NOR90, anti-Th/To and anti-PDGFR antibodies. MSA and MAA testing included assessment for presence of anti-Jo-1, Mi2 alpha, Mi2 beta, PL-7, PL-12, EJ, OJ, SRP, TIF1γ, MDA5 NXP2 and SAE1 antibodies. CK, ESR and CRP were considered elevated if results were greater than the local laboratory reference range. All participants underwent sMRI of the thighs, cardiac magnetic resonance imaging (CMR) and respiratory function tests. Respiratory function tests of interest were forced vital capacity (FVC), measured as percent predicted.

### Skeletal muscle magnetic resonance imaging

All sMRI examinations were performed on a 3T scanner (Magnetom Prisma, Siemens Healthineers, Erlangen, Germany) with a quadrature body coil. Bilateral axial thigh images were acquired with two Siemens 18ch body array coils positioned on the anterior aspect of both legs above the patella and over the hip. The scanning protocol consisted of both a T1-weighted turbo spin echo sequence (34 slices, slice thickness 7.0 mm, repetition time 706.0 ms, 11 ms) and a T2-weighted sequence (34 slices, slice thickness 7.0 mm, repetition time 228.46 ms, echo time 1.25 ms, flip angle 12°).

Skeletal muscle images were reviewed independently by two experienced musculoskeletal radiologists (MP, WP) for evidence of muscle and fascial oedema using T2-weighted images. Presence of muscle atrophy and fatty infiltration was evaluated using T1-weighted images. The presence of muscle atrophy and/or fatty infiltration, in excess of what would be expected for patient age and sex, was considered indicative of chronic muscle damage. Images were graded on a semi-quantitative scale, 0 = unaffected, 1 = mild change, 2 = moderate change and 3 = severe change. Disagreements were resolved by consensus. All skeletal muscle imaging was reviewed blinded to clinical information regarding disease duration, SSc manifestations, muscle strength or other investigation results.

Skeletal muscle T2-mapping times were calculated for each patient using Circle CV142 (Circle Cardiovascular Imaging, Calgary, Canada). Four muscle compartments of the thigh were evaluated: gluteal (gluteus maximus, quadratus femoris, obturator externus), quadriceps (rectus femoris, vastus lateralis, vastus medialis, vastus intermedius, sartorius), adductor (adductor brevis, adductor longus, adductor magnus, gracilis, pectineus) and hamstrings (biceps femoris (long head and short head), semitendinosis, semimembranosus). At least two regions of interest were manually drawn for each muscle from distal and proximal slices. Mean T2-mapping times for each compartment were derived from the mean of individual muscle scores, and an overall mean T2-mapping time across all compartments was calculated for each patient. Mean T2-mapping times were compared to the radiologists’ oedema scores for each compartment, as well as overall mean T2-mapping time to the presence of any muscle oedema in any muscle compartment detected by visual assessment.

Skeletal muscle volume and FF calculations were performed using SliceOmatic (v. 5.0, Tomovision, Magog, Canada). Analysis was conducted on eight consecutive slices, starting distally from the insertion of adductor longus muscle. Bone, skeletal muscle and adipose tissue were defined using signal intensity mapping on in-phase images. Adipose tissue was further divided into two categories: subcutaneous, and inter- and intramuscular adipose tissue. The defined regions of skeletal muscle and inter- and intramuscular adipose tissue were compared to fat enhanced images and the regions were manually adjusted, where disparities between in-phase and fat enhanced images existed. Bilateral skeletal muscle, and inter- and intramuscular adipose tissue volumes across the eight slices were calculated based on the surface area across each slice for each tissue type and multiplied by the slice thickness. A mean percentage FF for each patient was calculated by dividing the volume of inter- and intramuscular adipose tissue with the sum of inter- and intramuscular adipose tissue and skeletal muscle volumes across the eight analysed slices. FF was compared to visually assessed muscle damage, indicated by the presence of either muscle atrophy or fatty muscle infiltration.

### Cardiac evaluation

All CMR examinations were performed on a 3T scanner (Magnetom Prisma, Siemens Healthineers, Erlangen, Germany). All image post processing was performed using a dedicated software package (CVI42, Circle Cardiovascular imaging, Calgary, Canada).

### Assessment of cardiac function

Three long-axis and a contiguous short-axis stack of cine images (8-mm slice thickness, no gap) were acquired using an ECG-gated balanced steady-state free precision (SSFP) sequence in expiration. Left ventricular (LV) mass, end-diastolic volume, end-systolic volume and LV ejection fraction (LVEF) were quantified using CVI42 using a summation of disk method. Papillary muscles were regarded as part of the ventricular cavity. Measurements were indexed to body surface area.

### Evaluation of global longitudinal strain

Global longitudinal strain (GLS) was calculated using feature tracking on the cine images on CVI42 software. The myocardium was defined according to American Heart Association segments by placing a marker across the mitral valve annulus and from the annulus to the apex on long axis images and by marking endocardial and epicardial borders in the short axis volumetric stack and three apical cine images (4 chamber, 2 chamber, 3 chamber). Markers were placed at both right ventricle insertion points on the short axis images. The feature-tracking algorithm within the CVI42 software calculated GLS.

### Assessment of diffuse myocardial fibrosis

Native T1 (pre-contrast) and post contrast T1 times were measured in the myocardium and left ventricular blood pool using a region of interest on the T1 pixel map. T1 measurements were taken at the mid SAX level, both by including the entire myocardium (excluding artefact) and away from the blood pool. Myocardial T1 times were derived using the saturated recovery single-shot acquisition (SASHA) and shortened modified look locker (ShMOLLI) sequences which automatically generated pixel maps of T1 times. These were used during post-processing with a motion correction algorithm applied to the raw images. Post-contrast sequences were analysed 10 minutes following intravenous bolus injection of gadolinium-diethylene triamine penta-acetic acid (0.2 mmol/kg BW, Magnevist, Schering, Germany). Patient T1-mapping times were compared to the normal reference values established using our facility’s scanner and analysis techniques [30].

### Assessment of focal myocardial fibrosis

Regional myocardial fibrosis was visually identified by delayed enhancement within the myocardium, defined as areas of increased signal intensity post contrast when compared to nulled healthy myocardium. T1 measurements were taken within a region of the septum. Any mid-wall fibrosis (typical of dilated cardiomyopathy) was included, with the consideration that this represents a continuum with diffuse interstitial fibrosis.

### Assessment of myocardial oedema

Myocardial T2 times were measured using a T2-prepared fast angle low shot (FLASH) sequence that acquires three single-shot T2-weighted images in the same diastolic phase, each with a different T2 preparation time (preparation times 0 ms, 25 ms, 55 ms). A non-rigid image registration algorithm is used for in-plane motion correction before subsequent pixel-wise fitting of a two-parameter equation assuming a mono-exponential T2 signal decay. T2 measurement was taken within a region of the septum at the mid SAX level. CMR images were analysed by BC and ALG, blinded to any clinical information.

### Statistical analysis

Categorical variables are presented as number (percentage) and continuous variables as mean (± standard deviation (SD)) or median (interquartile range (IQR)), as appropriate. The characteristics of patients with and without skeletal muscle oedema, as defined by radiologist assessment, were compared using the chi-square test for categorical variables the independent two-sample *t*-test or Wilcoxon rank-sum test for continuous variables, as appropriate. Linear regression analysis was used to assess the relationship between quantitative sMRI and (i) clinical characteristics and (ii) visually assessed muscle oedema and fatty infiltration.

Study data were collected and managed using REDCap electronic data capture tools hosted at The University of Melbourne. Statistical analysis was performed using STATA 15.1 (StataCorp, College Station, TX, USA).

## Results

Thirty-four patients were recruited to this study. One patient was lost to follow-up prior to completion of investigations; another was unable to tolerate sMRI due to claustrophobia. There were 32 sMRIs available for analysis and 31 CMR; one CMR was not interpretable due to a high burden of ventricular ectopics. One patient did not receive gadolinium contrast during CMR. Population characteristics are detailed in Table 1.

**Table 1:** Population characteristics

	*Entire population (n = 32)*	*T2 hyperintensity (n = 13)*	*No T2 hyperintensity (n = 19)*	*p value*
Age (years) (mean, SD)	55.47 (7.71)	55.69 (8.36)	55.32 (7.47)	0.89
Female (*n*, %)	23 (71.88%)	7 (53.7%)	16 (84.2%)	0.06
dcSSc (*n*, %)	19 (59.38%)	12 (92.3%)	7 (36.8%)	< 0.01
Disease duration (years) (median, IQR)	10.17 (2.38–12.42)	11.17 (2.46–15.34)	10.16 (2.38–12.3)	0.79
Disease duration < 4 years (*n*, %)	13 (40.62%)	5 (38.5%)	8 (42.1%)	0.84
*Autoantibodies*^*a*^				
Anticentromere (*n*, %)	8 (25.00%)	3 (23.1%)	5 (26.3%)	0.84
Scl-70 (*n*, %)	12 (37.50%)	5 (38.5%)	7 (36.8%)	0.93
RNA polymerase III (*n*, %)	4 (12.50%)	3 (23.1%)	1 (5.3%)	0.14
Jo-1 (*n*, %)	1 (3.13)	1 (7.7%)	0 (0%)	0.22
PMScl (*n*, %)	3 (9.68%)	1 (8.3%)	2 (10.5%)	0.84
PL-12^b^ (*n*, %)	2 (8.33%)	1 (50%)	1 (50%)	0.23
*Disease manifestations*				
mRSS (median, IQR)	10 (3–18)	20 (15–29)	4 (2–10)	< 0.01
mRSS > 14 (*n*, %)	12 (37.50%)	10 (76.9%)	2 (10.5%)	< 0.01
ILD (requiring treatment) (*n*, %)	5 (15.62%)	2 (15.4%)	3 (15.8%)	0.98
FVC (% predicted) (median, IQR)	91 (77–98.5)	83.69 (22.34)	91.16 (18.61)	0.31
FVC < 70% (*n*, %)	6 (18.75%)	5 (38.5%)	1 (5.3%)	0.02
Digital ulcers	17 (53.12%)	8 (61.5%)	9 (47.4%)	0.43
Arthritis (*n*, %)	19 (59.38%)	8 (61.5%)	11 (57.9%)	0.84
Myositis^c^ (*n*, %)	2 (6.25%)	2 (15.4%)	0 (0%)	0.08
MMT score (median, IQR)	5 (5–5)	5 (5–5)	5 (5–5)	0.64
Chronic muscle damage on MRI^d^ (*n*, %)	6 (18.75%)	4 (30.77%)	2 (10.52%)	0.15
ESR (median, IQR)	15 (11–24.5)	15 (12–20)	15 (11–29)	0.79
CRP (median, IQR)	5 (4–7)	5.5 (4.5–9.5)	5 (4–7)	0.56
CK (median, IQR)	86 (69.5–110)	94 (71–111)	85 (68–102)	0.94
*Cardiac magnetic resonance imaging*				
LVEF (%) (mean, SD)	64.39 (6.37)	63.67 (5.84)	64.84 (6.80)	0.63
LV GLS (%) (mean, SD)	− 16.84 (2.31)	− 16.55 (2.78)	− 17.03 (2.00)	0.58
T2-mapping (FLASH) (ms) (mean, SD)	42.48 (3.74)	42.56 (4.21)	42.43 (3.54)	0.93
T1-mapping (SASHA) (ms) (mean, SD)	1583.81 (46.24)	1599.46 (45.47)	1573.93 (45.09)	0.14
T1-mapping (ShMOLLI) (ms) (mean, SD)	1218.47 (38.71)	1232.35 (33.85)	1209.71 (39.85)	0.11
LGE (*n*, %)	9 (30.00%)	5 (41.67%)	4 (22.2%)	0.26

*Abbreviations: CK* creatine kinase, *CMR* cardiac magnetic resonance imaging, *CRP* C-reactive protein, *dcSSc* diffuse cutaneous systemic sclerosis, *ESR* erythrocyte sedimentation rate, *FVC* forced vital capacity, *GLS* global longitudinal strain, *ILD* interstitial lung disease, *IQR* interquartile range, *LGE* late gadolinium enhancement, *LV* left ventricle, *LVEF* left ventricular ejection fraction, *MMT* manual muscle testing, *MRI* magnetic resonance imaging, *mRSS* modified Rodnan skin score, *SD* standard deviation, *Scl-70* anti-topoisomerase I antibody, *TTE* transthoracic echocardiogram, *T2* hyperintensity positive T2 signal on MRI indicating muscle oedema

^a^ Results shown for only those antibodies that were positive in at least one patient

^b^ 24 patients had PL-12 antibody testing

^c^ Clinically diagnosed elevated CK and evidence on MRI or muscle biopsy

^d^ Evidence of either muscle atrophy or fatty infiltration of muscle based on qualitative assessment of muscle by radiologists

Six patients (18.75%) patients had an elevated CK (range 145–622 IU) and four (12.50%) had weakness on MMT. Two patients (6.25%) had concurrent proximal weakness and elevated CK. Two patients had previously received treatment for myositis, but their muscle disease was considered clinically quiescent at the time of sMRI.

In total, 13 patients (40.62%) had evidence of muscle oedema on sMRI. Only two such patients had weakness and one had an elevated CK. There was no significant relationship between muscle oedema and serum CK (*p* = 0.94) or inflammatory markers (ESR: *p* = 0.79; CRP: *p* = 0.56). The two patients with a history of myositis both had muscle oedema on sMRI.

Six patients (18.75%) had evidence of muscle damage. One patient (3.13%) had mild, unilateral muscle atrophy and five patients (15.63%) had fatty muscle infiltration which was bilateral in 60% of cases. There was no relationship between chronic muscle changes and clinical weakness (*p* = 0.73). Muscle oedema was present in 4/5 (80.00%) patients with fatty muscle infiltration, suggesting concurrent acute and chronic muscle changes.

### Patterns and severity of muscle involvement

**Fig. 1:**
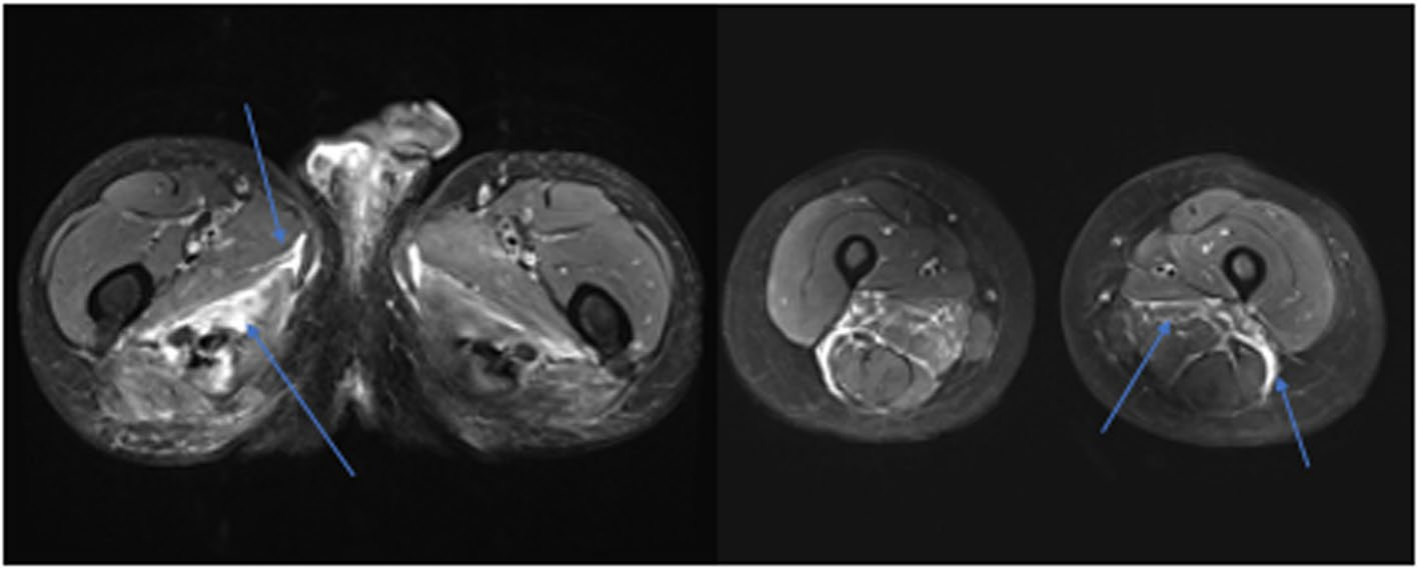
Muscle and fascial oedema in systemic sclerosis myopathy. Areas of muscle and fascial enhancement highlighted by blue arrows

Muscle oedema was most commonly observed in the quadriceps (*n* = 9, 69.23%) and gluteal muscles (*n* = 7, 53.85%). The adductor compartment was involved in five patients (38.46%) and the hamstrings in four (30.77%). Seven patients (53.85%) had moderate or severe oedema and notably six such patients had concurrent fascial oedema (see Fig. 1). Patients with severe muscle oedema had multiple muscle groups involved. No patient had isolated fascial oedema.

### Quantitative skeletal muscle MRI findings

Higher skeletal muscle T2-mapping times were associated with proximal muscle weakness (*p* < 0.001). No other clinical parameter was significantly associated with skeletal muscle T2-mapping time. Notably, CK and inflammatory markers were not significantly associated with T2-mapping times.

Percentage FF was negatively associated with serum CK (*p* = 0.01) but no other clinical parameter. Lower muscle volumes were associated with reduced strength (*p* = 0.012), and a trend was observed with reduced FVC (*p* = 0.083).

**Fig. 2:**
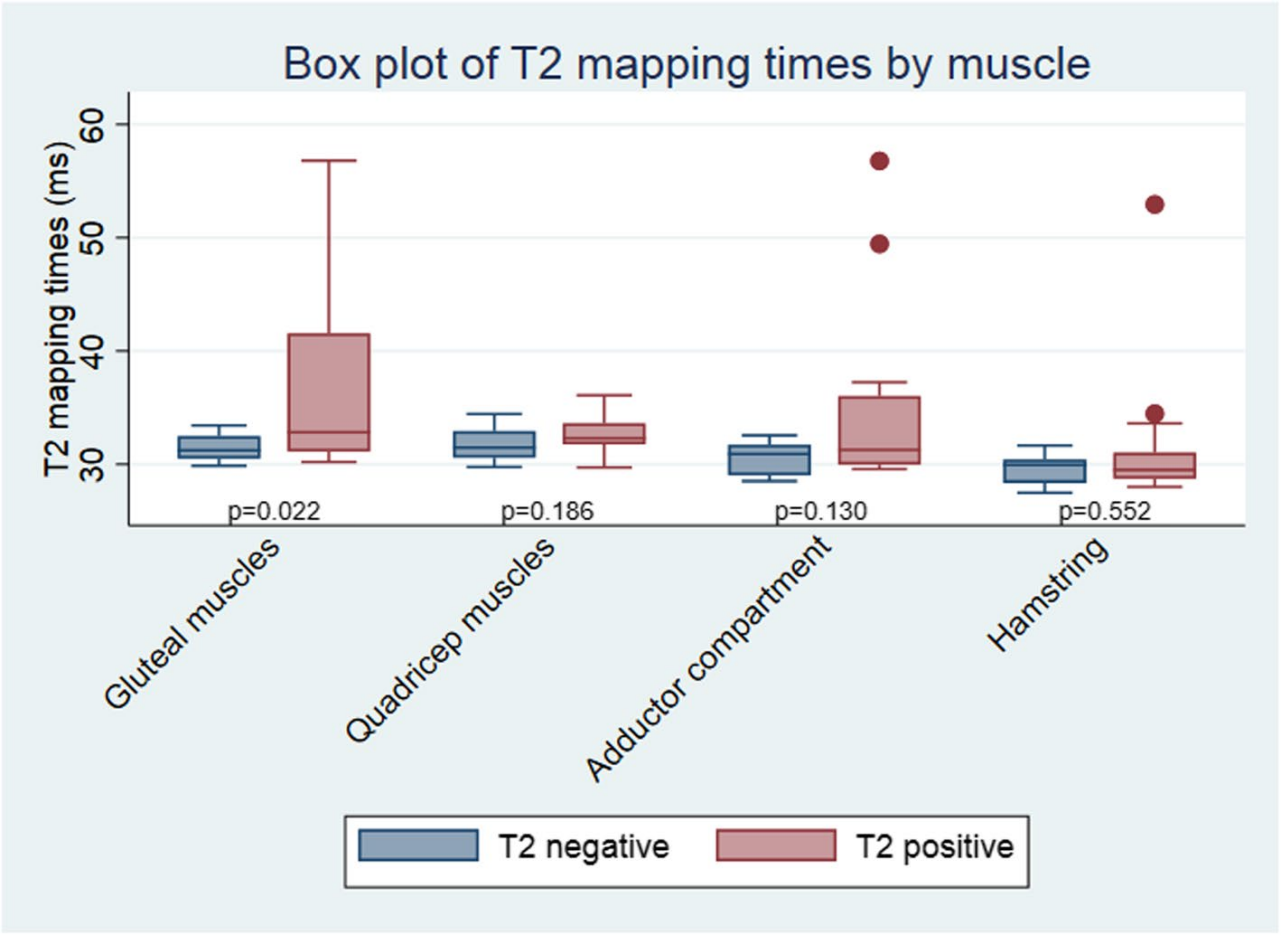
Comparison of T2-mapping times to radiologist-assessed muscle oedema. T2 negative: normal T2 signal on MRI indicating no oedema; T2 positive: T2 hyperintensity recorded indicating muscle oedema

Mean T2-mapping time, considered a measure of oedema or inflammation, was significantly associated with the presence of visually assessed muscle oedema, fatty infiltration and evidence of chronic muscle disease (see Table 2). The association between T2-mapping time of individual muscle compartments and radiologist assessed oedema was compared and findings are summarised in Fig. 2. Percentage FF was not significantly associated with presence of visually assessed fatty infiltration.

**Table 2:** Clinical and imaging associations of quantitative analysis of skeletal muscle changes

	*Muscle inflammation*		*Muscle damage*			
	*Mean T2-mapping time*		*Fat fraction*		*SMT*	
*Visual imaging assessment*	Coefficient (95% CI)	*p* value	Coefficient (95% CI)	*p* value	Coefficient (95% CI)	*p* value
Muscle oedema^a^	2.46 (0.44–4.48)	0.019	− 2.16 (− 5.17 to 0.85)	0.153	− 67.99 (− 286.11 to 150.13)	0.529
Fatty infiltration	3.41 (0.69–6.13)	0.016	− 3.28 (− 7.31 to 0.75)	0.107	− 128.46 (− 421.60 to 164.67)	0.378
Any chronic muscle damage on MRI^b^	3.03 (0.48–5.59)	0.022	− 2.32 (− 6.14 to 1.50)	0.224	− 149.02 (− 419.69 to 121.64)	0.279
*Clinical variable*						
Age	0.25 (− 0.12 to 0.17)	0.728	− 0.07 (− 0.27 to 0.13)	0.482	− 4.28 (− 18.39 to 9.84)	0.541
mRSS	0.05 (− 0.05 to 0.15)	0.315	− 0.02 (− 0.17 to 0.12)	0.754	− 2.61 (− 12.77 to 7.56)	0.605
mRSS > 14	1.73 (− 0.42 to 3.89)	0.111	− 1.34 (− 4.46 to 1.78)	0.387	− 39.96 (− 262.22 to 182.31)	0.716
Disease duration	0.05 (− 0.11 to 0.20)	0.546	0.01 (− 0.20 to 0.22)	0.934	− 12.72 (− 27.33 to 1.90)	0.086
Current prednisolone use	0.41 (− 1.94 to 2.75)	0.727	− 2.36 (− 5.54 to 0.82)	0.140	46.67 (− 185.34 to 278.69)	0.684
MMT < 5	− 5.58 (− 8.14 to 3.02)	< 0.001	1.44 (− 3.15 to 6.04)	0.526	382.12 (88.80 to 675.44)	0.012
CK	0.003 (− 0.007 to 0.01)	0.602	− 0.02 (− 0.03 to − 0.01)	0.006	0.94 (− 0.03 to 1.91)	0.057
ESR	− 0.06 (− 0.14 to 0.01)	0.079	0.002 (− 0.11 to 0.11)	0.970	2.42 (− 5.10 to 9.94)	0.516
CRP	0.02 (− 0.21 to 0.24)	0.879	0.12 (− 0.18 to 0.41)	0.414	0.39 (− 21.65 to 22.43)	0.971
Arthritis	− 2.00 (− 4.09 to 0.09)	0.060	1.12 (− 1.97 to 4.20)	0.465	87.97 (− 129.15 to 305.09)	0.415
Digital ulcers	− 0.60 (− 2.77 to 1.57)	0.576	− 0.29 (− 3.35 to 2.78)	0.850	9.21 (− 206.88 to 225.29)	0.931
ILD	− 0.94 (− 3.93 to 2.04)	0.523	− 1.68 (− 5.84 to 2.49)	0.417	198.43 (− 89.22 to 486.09)	0.169
FVC < 70%	− 1.94 (− 0.76 to 4.64)	0.152	2.14 (− 1.70 to 5.98)	0.264	230.71 (− 31.87 to 493.28)	0.083
*Cardiac imaging*						
LVEF (CMR)	− 0.05 (− 0.22 to 0.13)	0.606	0.18 (− 0.06 to 0.43)	0.131	− 8.41 (− 25.84 to 9.02)	0.332
LV GLS (CMR)	0.15 (− 0.35 to 0.65)	0.545	− 0.60 (− 1.30 to 0.09)	0.085	18.44 (− 31.38 to 68.27)	0.455
T1-mapping (SASHA)	0.04 (0.02 to 0.06)	0.001	0.005 (− 0.03 to 0.04)	0.780	− 1.78 (− 4.13 to 0.56)	0.131
T1-mapping (ShMOLLI)	0.02 (− 0.01 to 0.05)	0.117	0.02 (− 0.02 to 0.06)	0.261	− 0.72 (− 3.63 to 2.18)	0.614
T2-mapping (FLASH)	0.23 (− 0.06 to 0.52)	0.118	0.11 (− 0.32 to 0.54)	0.603	− 28.41 (− 56.57 to − 0.25)	0.048

Analysis by linear regression

*Abbreviations: CI* confidence interval, *CK* creatine kinase, *CMR* cardiac magnetic resonance imaging, *FVC* forced vital capacity, *GLS* global longitudinal strain, *ILD* interstitial lung disease, *LV* left ventricle, *LVEF* left ventricular ejection fraction, *MMT* manual muscle testing, *MRI* magnetic resonance imaging, *mRSS* modified Rodnan skin score, *SMT* skeletal muscle tissue

^a^ Muscle oedema present based on qualitative assessment of muscle by radiologists

^b^ Evidence of either muscle atrophy or fatty infiltration of muscle based on qualitative assessment of muscle by radiologists

### Myositis specific autoantibodies

MAA known to be associated with SSc and Jo-1 were tested in all patients. Extended MSA and MAA testing was performed in 26/32 (81%) patients. Overall, 7 patients (21.88%) had myositis-associated autoantibodies present. Two patients (7.69%) were PL-12 positive with no clinical features suggestive of anti-synthetase syndrome. One patient had dual positivity for anti-Jo-1 and anti-PL-12. Neither patient with PL-12 positivity had evidence of skeletal muscle oedema. One patient (3.85%) was Mi2 alpha positive. This patient had no evidence of muscle inflammation or cutaneous manifestations associated with dermatomyositis but had severe interstitial lung disease. Three patients (9.38%) were PMScl positive; only one had muscle oedema on sMRI.

### SSc-associated myopathy and systemic sclerosis heart involvement

Diffuse myocardial fibrosis, measured by native T1-mapping time, was highly prevalent. One-third (*n* = 9) of the study population had focal areas of late gadolinium enhancement. 29/31 (93.55%) had elevated native T1 times measured by SASHA sequences and 30/31 (96.77%) had elevated native T1 times measured by ShMOLLI sequences [30]. Mean T2-mapping time, measuring diffuse myocardial inflammation, was 42.48 ms±3.74 ms (normal value 35 ms).

No parameter of LV function was associated with radiological features of SM. There was no association between SM and diffuse myocardial inflammation (*p* = 0.118). The relationship between cardiac findings and radiologist assessment of skeletal muscle are detailed in Table 1 and quantitative parameters in Table 2.

## Discussion

In a cohort of SSc patients, unselected for musculoskeletal involvement, we detected a high prevalence of muscle abnormalities using sMRI. This is the first study to evaluate the role of T2-mapping and FF in SM. Evidence of the construct and criterion validity of T2-mapping and FF in IIM is emerging [25–27]. In this study, T2-mapping times were associated with radiologist-assessed presence of muscle oedema, providing initial support for the use of quantitative sMRI sequences in the assessment of SM.

There are limited published data describing the pattern of skeletal muscle involvement in SSc. A small series of 18 patients with musculoskeletal symptoms documented evidence of myositis in 79% of patients, generally in a symmetrical distribution [14]. We have observed preferential involvement of gluteal and quadriceps muscles, consistent with a recent retrospective analysis of MRI findings in SSc patients with clinical weakness [31]. T2-mapping times were observed to be highest in the gluteal muscle compartment, suggesting that oedema may be most severe in this muscle group. This may explain why when considering T2-mapping times in individual muscle compartments, a significant difference was only observed in the gluteal muscle group. The small number of patients with positive findings in each of the individual muscle groups limits the statistical power of any comparison of T2-mapping times, highlighting the need for larger studies to map the patterns of myopathy in SSc. In light of our study results, it is reasonable to hypothesise that future studies may be able to determine significant differences in the T2-mapping time between individual muscle compartments and potentially quantify the severity of involvement of specific muscle groups.

Musculoskeletal symptoms of SSc are associated with poorer quality of life and muscle weakness is linked to reduced physical function [32, 33]. The underappreciated burden of SM is likely to be ‘silently’ contributing to the fatigue and functional impairment commonly observed in SSc [34, 35]. Clinical weakness, serum CK and inflammatory markers are poorly sensitive tests of active SM. Histopathological changes in SSc muscle are not universally inflammatory, with a high frequency of muscle necrosis, fibrosis, atrophy and vasculopathic changes seen [4]. CK is released as a result of excessive sarcoplasmic membrane permeability [36], and histological inflammation or necrosis is associated with higher CK levels in SM [5]. The high frequency of non-inflammatory and fibrosing myopathy observed in SSc may account for the lack of association between sMRI changes and serum CK and inflammatory markers. The asymptomatic muscle involvement detected in this study highlights the need to consider imaging and or histopathological assessment to fully evaluate patients for possible SM. Accurate ascertainment of the burden and subtype of muscle involvement is of clinical importance as inflammatory muscle disease may be reversible with immunosuppression. Clinical experience with IIM indicates that treatment and adequate suppression of inflammation improves patient outcomes, reducing long-term disability [37]. There is much interest in the role of exercise therapy as a treatment modality of other myopathies, which remains untested in SM. There is no robust data to support treatment recommendations for the management of SM particularly if it is a non-inflammatory subtype. However, given the potential functional gains that could be made with effective treatment for SM, testing of both pharmacological and non-pharmacological therapies for SM should be considered a high research priority.

Muscle oedema was more commonly found in men and those with dcSSc. The frequency of SM did not differ according to SSc disease duration. SM has been associated with anti-PMScl, anti-Ku, anti-U1RNP, Th/To and RuvBL1/2 autoantibodies [4]. Whilst seven patients had detectable autoantibodies associated with muscle disease in this study, we did not find any clear associations between muscle oedema and autoantibody status; however, our study population was too small to detect significant associations with uncommon autoantibodies. We investigated the link between cardiac and skeletal muscle involvement. Given the important association between heart involvement, risk of heart failure and sudden cardiac death, an association with skeletal muscle involvement could be clinically helpful. It is important to know whether the finding of SM should be a trigger for more extensive cardiac assessment. However, in this study, skeletal muscle oedema had no relationship with parameters of cardiac function or myocardial inflammation. Whilst this is a small study and likely underpowered to determine the association between imaging findings and clinical outcomes, we have demonstrated that there are significant abnormalities of both skeletal and cardiac muscle compared to normal values. These results demonstrate the capacity for advanced imaging to be used as a tool in longitudinal studies to better understand any potential mechanistic link between skeletal myositis and cardiac complications of SSc and identify subtle pathology prior to the onset of symptoms.

This study is limited by a lack of histopathological correlation with imaging findings. There are conflicting reports as to the correlation of sMRI findings with histopathological subtypes of SM [5, 31], and no conclusions can be drawn as to the underlying histopathological mechanisms of the abnormalities detected in this study. The small study population precludes making definitive conclusions about patterns of muscle involvement and clinical correlations of imaging abnormalities. Future studies may seek to define the radiological pattern of SM and determine whether imaging can help distinguish inflammatory from fibrotic subtypes of muscle involvement. Importantly, this is the first study to systematically assess the skeletal muscles of SSc patients, unselected for burden of musculoskeletal symptoms or proximal weakness.

## Conclusions

SM is an important and underappreciated disease manifestation that is associated with poor function and increased mortality [5, 6]. sMRI can reveal a high burden of skeletal muscle involvement that is independent of commonly used biomarkers of muscle disease, namely MMT, CK and inflammatory markers. Patients with SSc commonly report reduced function, poor exercise tolerance, patient-perceived muscle weakness and pain from very early in the disease course [38, 39]. SM is a potentially reversible disease manifestation that is likely contributing to these symptoms. SSc cardiac involvement is also highly prevalent but does not appear to have a relationship with skeletal myopathy in the absence of clinical symptoms of cardiac disease.

Further studies should establish whether a combination of imaging and serum biomarkers can predict SM and further define the role of imaging in combination with histopathology in confirming a diagnosis of SM and the monitoring disease progression. Importantly, future research should investigate whether more aggressive treatment of myopathy results in improved function, quality of life and survival for patients with SSc.

## Data Availability

The datasets generated and analysed during the current study are not publicly available due to protection of individuals’ privacy but are available from the corresponding author on reasonable request.

## Abbreviations

CK: Creatine kinase
CMR: Cardiac magnetic resonance imaging
CRP: C-reactive protein
dcSSc: Diffuse cutaneous systemic sclerosis
ECG: Electrocardiograph
ECV: Extra cellular volume
ESR: Erythrocyte sedimentation rate
FF: Fat fraction
FLASH: Fast angle low shot
FVC: Forced vital capacity
GLS: Global longitudinal strain
IIM: Idiopathic inflammatory myopathy
IQR: Interquartile range
LV: Left ventricle
LVEF: Left ventricular ejection fraction
MAA: Myositis associated antibody
MMT: Manual muscle testing
mRSS: Modified Rodnan skin score
MSA: Myositis specific antibody
SASHA: Saturated recovery single-shot acquisition
SD: Standard deviation
ShMOLLI: Shortened modified look locker
SM: Systemic sclerosis associated myopathy
sMRI: Skeletal muscle magnetic resonance imaging
SRP: Signal recognition particle
SSc: Systemic sclerosis
STIR: Short tau inversion recovery
